# Parallel Generalized Real Symmetric-Definite Eigenvalue Problem

**DOI:** 10.6028/jres.125.032

**Published:** 2020-11-06

**Authors:** James S. Sims, María Bélen Ruiz

**Affiliations:** 1 National Institute of Standards and Technology, Gaithersburg, MD 20899, USA; 2 Friedrich-Alexander-University Erlangen-Nürnberg Egerlandstraße 3, D-91058, Erlangen, Germany

**Keywords:** dense matrix eigensolver, high precision, parallel

## Abstract

A computationally fast Fortran 90+ quadruple precision portable parallel GRSDEP (generalized real symmetric-definite eigenvalue problem) package suitable for large (80,000 x 80,000 or greater) dense matrices is discussed in this paper.

**Software DOI:** https://doi.org/10.18434/mds2-2293

## 1 Introduction

Few-electron atomic and molecular systems continue to play an important role as a testbed for understanding fundamental physical and chemical theories. Non-relativistic ground state energies and wave functions for few-electron atoms are primary inputs for computations of atomic properties such as electron affinities and ionization potentials, as well as for computing perturbative corrections due to relativity and quantum electrodynamics [1]. Furthermore, fundamental quantum theory dictates that, in general, uncertainties in wave functions scale as the square root of uncertainties in corresponding energies. Therefore, when using convergence of energies as a metric for convergence of associated wave functions, very high accuracy in the former must be achieved to guarantee accuracy in the later.

To achieve this high accuracy (real(24) arithmetic is necessary for 2 electron systems and real(16) for more than 2 electron systems) requires explicitly correlated wave functions which in turn lead to dense matrices with sizes (N) that are large compared to most dense matrix problems. This in turn leads to linear dependency issues that are solved by going to higher precision, all of which means that calculations need to be parallelized for both computational speed and to spread the memory out across a cluster. Hence parallel packages like ScaLAPACK, which is available only in real(8), cannot be used as they stand and the packages are too big to contemplate conversion to real(16). This has led to the development of the portable parallel GRSDEP (generalized real symmetric-definite eigenvalue problem) package which is discussed in this paper.

## 2 Parallel GRSDEP

Computational Quantum Chemistry involves solving SchrÖdinger's equation for *N_e_* electrons

*H*ψ(**X**_1_, **X**_2_, *...,* **X***_Ne_*) = *E*ψ(**X**_1_, **X**_2_, *...,* **X***_Ne_*), (1)

where **X_i_** = (**r_i_**, *ξ***_i_**) is the combined space-spin coordinate for electron i, ψ(**X**_1_, **X**_2_, *...,* **X***_Ne_*) represents a wave function describing a given quantum mechanical system, E is the energy of the system, and *H* is the Hamiltonian operator specifying components of the energy included in the model.

The wave function given by Equation (1), ψ(**X**_1_, **X**_2_, *...,* **X***_Ne_*), is a linear combination of terms Φ*_K_*, where the coefficients *c_K_* are those which minimize the total energy, E, given by

$E=\frac{<\psi ℋ|\psi >}{<\psi |\psi >}=\frac{\Sigma_{K,L}c_{K}c_{L}H_{KL}}{\Sigma_{K,L}c_{K}c_{L}S_{KL}},$ (2)

where

*H_KL_* =< Φ*_K_* |*ℋ*|Φ*_L_ >*; *S_KL_* =*<*Φ*_K_*|Φ*_L_ > .* (3)

The condition for the energy to be an extremum, *δE* = 0, is the well-known matrix eigenvalue (secular) equation:

$\sum_{L=1}^{N}c_{L}H_{KL}=E\sum_{L=1}^{N}c_{L}S_{KL}$(4)

Solving this equation is equivalent to solving the *N*-dimensional generalized eigenvalue problem (GEVP)

**Hc** = *E***Sc**, (5)

where **H** and **S** are both symmetric, have matrix elements *H_KL_* and *S_KL_* given by Equation (3), *S* is positive definite and *N* is the number of configurations Φ*_K_* in ψ. Equation (5) has *N* solutions defined by the pairs {(*E,* **c**)*_i_, i* = 1, *N*}, each of them fulfilling the variational principle ([2]) for the *i*th quantum state of the system.

As the Hamiltonian operator *H* is hermitian, the matrices involved are symmetric (which makes this a generalized real symmetric-definite eigenvalue problem (GRSDEP)), so it is sufficient to compute only the upper or lower triangle of the matrix (an important CPU time and memory saving feature). For use in quantum chemical calculations^1^1With applicability in other fields, such as atomic, molecular and optical physics, as well. several iterative procedures have been devised for Configuration Interaction(CI) [3-5] and for molecular elliptic coordinate calculations [6]. As pointed out by Lüchow and Kleindienst [7], all of these show slow convergence in the more accurate explicitly correlated calculations and cannot be applied successfully if high precision, spectroscopic accuracy is desired. This slow convergence is probably due to the large off-diagonal matrix elements in the (dense) matrices. For high precision, real(16) arithmetic is necessary for molecules and N *>* 2 electron atoms, and real(24) arithmetic is necessary for 2 electron atoms. Hence parallel packages like ScaLAPACK, which is available only in real(8), cannot be used as they stand and the packages are too big to contemplate conversion to real(16). For these reasons a portable parallel GRSDEP package was developed.

### 2.1 Inverse Iteration

Inverse iteration (also called shifted inverse power method in this case) is one of the fastest methods for getting selected roots of the generalized eigenvalue problem, particularly if one has a pretty good idea of what the eigenvalue should be, as is the case in our calculations [8, 9]. When a good starting approximation for an eigenvalue is not available, one uses other methods such as Givens [10] to obtain the starting approximation.

Letting **A** = **H** *-E*_0_**S**, inverse iteration, developed first by Wielandt [11], is the power method [12] applied to **A^-1^** = (**H** *-E*_0_**S**)^-1^ in the generalized eigenvalue problem, where **A^-1^** is the inverse of *A*. *E*_0_ is some starting approximation for the eigenvalue of interest. If **x** is an eigenvector of **H** with associated eigenvalue E (so that **Hx** = *E***Sx**), then subtracting *E*_0_**S** from both sides,

(**H** *-E*_0_**S**)**x** = (*E -E*_0_)**Sx**, (6)

**x** = (*E -E*_0_)(**H** *-E*_0_**S**)*^-^*^1^**Sx**, (7)

(**H** *-E*_0_**S**)*^-^*^1^**Sx** = (*E -E*_0_)*^-^*^1^**x**. (8)

If **v**_0_ is not an eigenvector of **H** it can be expanded in the eigenvectors of **H** as follows

$v_{0}=\sum_{i=1}^{N}c_{i}x_{i}$(9)

i.e., **v**_1_ is the vector obtained by operating with **A**^-1^**S** on **v**_0_, **v**_2_ is obtained by operating with **A**^-1^**S** on **v**_1_, etc.

Letting **A** = **H** *-E*_0_**S**, the power method applied to **A**^-1^discussed in the Appendix) generates the sequence {**v***_n_*} recursively, using

**v***_n_*_+1_ = **A***^-^*^1^**Sv***_n_.* (10)

Equation (10) is the basic iteration formula. Since it is an inverse, the eigenvalue ultimately converged upon will be a LOWER bound to the exact eigenvalue of this state, as required.

The inverse of **A** is not actually computed (which would not be efficient). Rather, as explained in the next (sub)section, L (and D) are computed and stored in a efficient form for subsequent use in an **L***^T^* **D***^-^*^1^**L** operation on **Sv***_n_*.

The equation which is used (see Appendix) to monitor the convergence to the final eigenvalue E is

$E=E_{0}+\frac{v_{n+1}\cdot Sv_{n}}{v_{n+1}\cdot Sv_{n+1}}$(1)

The convergence of **v***_n_* to **x** is faster the closer the trial *E*_0_ is to *E*. Hence it is desirable to have a pretty good idea of what the eigenvalue is. It is generally sufficient to know the eigenvalue to 5 digits. When that is not the case, one can use NAG [13] or LAPACK [14] libraries with smaller expansions to get the starting value.^2^2When we have no "guess" for the desired eigenvalue, we use a truncated wave function and solve the eigenvalue problem by reduction of **S** to a unit matrix followed by solving the transformed **H** for the lowest few eigenvalues using Givens [15].

### 2.2 Strategy for using Inverse Iteration

The (efficient) strategy for the inverse iteration without actually calculating **A***^-^*^1^ = (**H** *-E*_0_**S**)*^-^*^1^ is as follows.

By construction a lower triangular matrix **L** such that **LA** = **U**, an upper triangular matrix is found. Right multiply by **L***^T^* , where **L***^T^* is the transpose of **L**, to get

**LAL***^T^* = **UL***^T^* = **LU***^T^* = **D**, (12)

where **D** is a diagonal matrix since **LAL***^T^* is symmetric. Here the facts that the set of upper (lower) triangular matrices is closed under multiplication and that the intersection of the set of upper triangular matrices and lower triangular matrices is the set of diagonal matrices are used. Now solve for **A** to get

**A** = **L***^-^*^1^**DL***^-T^* (13)

and

**A***^-^*^1^ = **L***^T^* **D***^-^*^1^**L** (14)

where we have used the fact that the inverse of the transpose is the transpose of the inverse. Instead of **A***^-^*^1^ in Equation (10) **L***^T^* **D***^-^*^1^**L** is used.

It remains only to show how to determine **L** and **D** in the transformation of **A** to diagonal form. **L** is assembled by construction, namely,

**L** = **L***_N_***L***_N-_*_1_*...***L**_3_**L**_2_ (15)

and

**A**_2_ = **L**_2_**A** (16)

**A**_3_ = **L**_3_**A**_2_ (17)

*...* (18)

**A***_k_* = **L***_k_***A***_k-_*_1_. (19)

**L**_2_ is the lower triangular matrix that sets the off-diagonal lower triangular elements of the second row of **A** to zero, **L**_3_ operating on **A**_2_ does a similar thing for the third row of **A**_2_, and so on. In general, **L***_k_* is a matrix with 1s along the diagonal and zeros everywhere else except the lower triangular off-diagonal elements of row k, which are given by

*-* **A***_k-_*_1_(*i, k*)*/***A***_k-_*_1_(*i, i*), *i* = 1, 2, *..., k -* 1; (20)

that is, the elements of the k-th column of **A***_k-_*_1_ divided by the corresponding diagonal elements.

Once **A***_k_* is obtained column k (above the diagonal) is no longer needed and the off-diagonal elements of row k of **L***_k_* can be safely stored there. Once **A***_N_* is obtained, the diagonal elements constitute the diagonal matrix **D**. For convenience **D** is then replaced by **D***^-^*^1^. Vector **A** is now what we call the implicit representation of **L**. There is no need to multiply out the **L**s (an Order *N*^3^ operation); each operation on **x** by an **L***_k_* is at most an order *N* operation.

Note that the basic operation (**A***_k_* = **L***_k_***A***_k-_*_1_) in forming the **A***_k_*s is the Fortran 90+ DOT PRODUCT intrinsic function, which hopefully will be implemented efficiently. **L** = **L***_N_***L***_N-_*_1_*...***L**_3_**L**_2_ also involves only the DOT PRODUCT. **L***^T^* **y** (**y** = **D***^-^*^1^**L**) on the other hand uses the QAXPY BLAS operation, which also can be efficiently implemented for portability and extended precision.

A is not stored as an N by N array but rather as a vector of upper triangular columns, which are efficiently accessed by the DOT PRODUCT function (and similarly for QAXPY).

One thing to note is that with the large matrices that one deals with in parallel problems, memory access patterns can be extremely important. The initial coding got very slow as large *N* was approached. An essentially trivial reordering of the operations involved with the **L**s fixed the problem (a ROW form of **L** and a COLUMN form for **H** and **S** are now used) and led to substantial speedups for the large *N* case. All of which emphasizes that the construction of **L** is by no means unique and that this one is a very good one.

### 2.3 Parallelization

To parallelize one simply maps **A** to the several processors. A cyclic range is used to distribute elements of each row of the matrix **A** over the processors in a cluster. Each column is stored in its entirety on a particular processor. A cyclic "round robin" assignment of columns to the processors is made, which yields an approximately equal distribution of matrix elements to processors. This insures good load balancing (assuming homogeneous processors) and produces better load balancing than a block assignment (as in ScaLAPACK [16]) would.

In the parallel version of GRSDEP, MPI [17] is used to run the same program on multiple processors (on the same or different hosts) and each processor generates **H** and **S** only for the columns of **H** and **S** it owns.

The steps of the (parallel) inverse iteration are

1. Calculate **A** = **H** *-E***S**.

On each processor, calculate the **A**s for the columns of the matrices that belong to this processor.

2. Calculate **Sv_0_** using the vector **1**=(1,1,...,1) as the starting vector **v**_0_.

On each processor, calculate the partial vectors **b***_i_* = **S***_i_ ·* **1** (a row sum, **1** is a vector of all ones) and send them to the root process (process 0). Process 0 then sums them up to get **b** = **S** *·* **1**(= **Sv**_0_).

3. Find the transformation of **A** to the implicit representation of **L**.

Calculate the partial **L**s of **A** with the partial **D**s stored in the diagonal elements. Set *k* = 0.

4. Begin iteration.

(a) If (*n ≠ *0) Calculate **b***_n_* = **Sv***_n_*.

(b) Solve **v***_n_*_+1_=**L***^T^* **D***^-^*^1^**Lb***_n_* (Call Ltdm1L to do **L***^T^* **D***^-^*^1^**Lb***_n_*, returning the result as **x** which is **v***_n_*_+1_).

5. Calculate *E* = *E*_0_ + $\frac{v_{n+1}\cdot Sv_{n}}{Sv_{n+1}\cdot v_{n+1}}$.

6. Stop condition fulfilled?

(E changed negligibly between last two iterations?)

NO: set **v***_n_*_+1_ = ^$\frac{v_{n+1}}{r}$^where *r* =$\sqrt{v_{n+1}\cdot Sv_{n+1}}$; *n ← n* + 1; go to 4 (a).

YES: stop.

One other point to discuss is how to handle the **L**s and **D**s. Do we keep them distributed during the operation with **L** on **x**, or are the distributed **L**s gathered into processor 0 and the **L** operations carried out on a single processor (that is serially; but even with multiple processors the operation by **L** is still strictly serial). The problem with keeping **L** on a single processor is that it unbalances the memory requirements. So **L** (on top of **A**) is kept distributed and the vectors are passed to the processors in a round-robin fashion. So for the **Lx** operation

1. process 0 applies its **L**s to **x** and passes the resulting vector to process 1.

2. process 1 applies its **L**s to the **x** from process 0, then passes result to process 2.

3. process 2 applies its **L**s to the vector received from process 0 and so on.

Except for root (*P*_0_) a processor *P_i_* receives an **x** from *P_i-_*_1_ and sends the **x** it calculates to *P_i_*_+1_, except if *i* + 1 is greater than last, in which case **x** will be sent to *P*_0_, which will already have issued a receive. All processors except *P*_0_ start out with a receive from processor myrank - 1 and wait until a message arrives. For **L^T^x** one uses a similar logic except one starts with *P_last_* and works down to *P*_0_.

## 3 Summary of the Timing Tests

This section is an attempt to measure the effectiveness of the parallelization of the GRSDEP by calculating the time it takes (in seconds (s)) to compute the energy of a 4190 term *H*_2_ wave function with a resultant total energy of -1.1744 7571 hartrees (Ha) [9] on differing numbers of processors. Table 1 gives the times (in seconds (s)). All results were obtained using real(16) precision (quadruple precision or QP, 128-bit, ≈ 32 digits) floating point arithmetic on the National Institute of Standards and Technology (NIST)'s 16908 processor Linux cluster. The Message Passing Interface (MPI) Standard [17] was used to parallelize the code.

**Table 1:** The time it takes to compute the ground state energy of the hydrogen molecule as a function of the number of processors utilized on a Linux cluster.

Number of Processors	LD time (s)	Ltdm1L time (s)	Total time (s)
1	1392	26	1428
2	697	26	728
4	351	26	380
8	177	26	205
16	89	26	117
32	46	26	73

The LD step in the table refers to the time it takes to find **L** and **D** and gives a speedup of 30 on 32 processors, which is excellent! The 1 to 32 run speeds up by a factor of 20, a 23 minute job completing in a little over a minute. While performance can depend on the amount of memory available, the size of the problem (*N*), how one calculates **L^T^**, how elements of **A** are stored, etc., the speedup is ultimately limited by the two sequential steps, calculating the matrix elements and the Ltdm1L (**L***^T^* **D***^-^*^1^**L**) step.

## 4 Conclusions

While the parallel GRSDEP has proven quite useful, having been utilized for investigating the following systems:

⏺ 2-electron molecule: ground state of *H*_2_,

⏺ 2-,3-, 4-, and 5-electron atoms and their ions,

and producing some of the lowest (i.e. most accurate) variational energies ever reported, the lack of pivoting in this solver needs to be discussed.

The solver (shifted inverse iteration) used is one of the fastest methods for getting selected roots of the generalized eigenvalue problem. The only computational problem is the computation of the implicit representation of **L**. The addition of partial and/or complete pivoting in the use of inverse iteration complicates the code as the matrices go from triangular to square and much of the speed advantage of inverse iteration is lost. Equation (10) is also

**Av***_n_*_+1_ = **Sv***_n_* (21)

which is the familiar linear system of the form **Ax** = **b** for which LAPACK has specific routines with pivoting. One of these which became available in version 3.5 utilizes the bounded Bunch-Kaufman (rook) pivoting algorithm [18] which is more accurate than partial pivoting, with comparable costs. Since LAPACK is available in Fortran, it was not hard to produce real(16) versions of those routines in place of step 3 and 4.b in the inverse iteration algorithm (sequential case) outlined in Section 2.3 above, and hence test the solver-produced results reported in, for example [19]. It should be noted that the Ritz estimate

$E=E_{0}+\frac{v_{n+1}\cdot Hv_{n+1}}{v_{n+1}\cdot Sv_{n+1}}$. (22)

is not used to monitor convergence to the final eigenvector E, rather it is Equation (11), derived in the Appendix, which is used in step 2.3.5. This has the advantage that the original *H* matrix does not need to be saved, freeing that storage space up to be overwritten by *L*, which then can be used to solve for a principal minor of dimension *N*' with very little additional CPU time.

In all cases the results reported in [19] were reproduced to all digits reported in that work. However it took 9.5 days to reproduce the result for the largest (*N* = 39,381) matrix (which took only 176 minutes on 120 processors), obviously not fast enough for a production code even for matrices of this size or smaller, much less for the sizes computed (up to *N ≈* 80 K) in earlier work [20]. Public domain codes are available for inverse iteration with partial and complete pivoting, but they do not meet the need for a parallel code which is fast, efficient, numerically stable, and of high enough precision (real(16)) to enable calculations which go significantly beyond the currently investigated sizes. A real(16) parallel version of the Rook pivoting algorithm, such as the one(s) of Strazdins [21], could greatly facilitate this endeavor.

## 5 Software Specifications

**Table:** 

**NIST Operating Unit**	NIST Information Technology, Applied and Computational Mathematics, Scientific Applications and Visualization
**Category **	parallel GEVP (Generalized Eigenvalue Problem)
**Targeted Users **	High precision atomic and molecular science
**Operating Systems**	All
**Programming Language **	Fortran 90+
**Inputs/Outputs **	See the HowTo included with the software: https://doi.org/10.18434/mds2-2293
**Documentation **	Included with the software: https://doi.org/10.18434/mds2-2293
**Disclaimer **	https://www.nist.gov/director/licensing

## 6 Appendix: The power method applied to A = H -*E*S

Letting **v**_1_ be the vector which results from applying **A***^-^*^1^**S** to **v**_0_,

**v**_1_ = (**H** -*E*_0_**S**)*^-^*^1^**Sv**_0_ = (**H** -*E*_0_**S**)*^-^*^1^$\sum_{i=1}^{N}c_{i}x_{i}$(23)

= $\sum_{i=1}^{N}c_{i}$(**H** *-E*_0_**S**)*^-^*^1^**Sx***_i_* = $\sum_{i=1}^{N}c_{i}$ (*E_i_ -E*_0_)*^-^*^1^**x***_i_.* (24)

Simliarly, let **v**_2_ be the vector which results from applying **A***^-^*^1^**Sv** to **v_1_**, and similarly for **v_3_**, etc.

**v**_2_ = (**H** *-E*_0_**S**)*^-^*^1^**Sv**_1_ = $\sum_{i=1}^{N}c_{i}$ (*E_i_ -E*_0_)*^-^*^2^**x***_i_* (25)

...

**v***_n_* = (**H**- *E*_0_**S**)*^-^*^1^**Sv***_n_*_-1_ = $\sum_{i=1}^{N}c_{i}$ (*E_i_ -E*_0_)*^-n^***x***_i_.* (26)

From Equation (26) it is clear that if (*E_k_ -E*_0_) is small, after a few iterations the right hand side will be proportional to the eigenvector **x***_k_* (all terms but the *i* = *k* term will be small), so that

**v***_n_ ≈ c_k_*(*E_k_ -E*_0_)*^-n^***x***_k_.* (27)

Setting *E_k_* = *E* and applying **A***^-^*^1^**S** again to **v***_n_*, one gets

**v***_n_*_+1_ = **A***^-^*^1^**Sv***_n_,* (28)

**v***_n_*_+1_ = *c_k_*(*E -E*_0_)*^-^*^(^*^n^*^+1)^**x***_k_,* (29)

**v***_n_*_+1_ = (*E -E*_0_)*^-^*^1^*c_k_*(*E -E*_0_)*^-n^***x***_k_,* (30)

**v***_n_*_+1_ = (*E -E*_0_)*^-^*^1^**v***_n_,* (31)

(*E -E*_0_)**v***_n_*_+1_ = **v***_n_.* (32)

Applying **S** to both sides and then taking the dot product of both sides with **v***_n_*_+1_,

(*E -E*_0_)**v***_n_*_+1_ *·* **Sv***_n_*_+1_ = **v***_n_*_+1_ *·* **Sv***_n_,*(33)

*$E-E_{0}=\frac{v_{n+1}\cdot Sv_{n}}{v_{n+1}\cdot Sv_{n+1}}$* (34)

*$E=E_{0}+\frac{v_{n+1}\cdot Sv_{n}}{v_{n+1}\cdot Sv_{n+1}}$.* (35)

Which is Equation (11).
